# Forty-Sixth Annual Report of the Commissioners in Lunacy for Scotland

**Published:** 1904-10-01

**Authors:** 


### FORTY-SIXTH ANNUAL REPORT OF THE COMMISSIONERS IN LUNACY FOR SCOTLAND.

This report, with its appendices, runs to 180 pages and is presented to his Majesty's Secretary of State for Scotland. As usual with these Scottish Blue Books it is a very interesting, if somewhat saddening, document. It would seem that on January 1st, 1903, Scotland had 16,658 lunatics known as such to the authorities, and that by the corresponding date of 1904 the number had risen to 16,894, showing an increase of 236 during the year. It is remarkable that the increase is relatively much higher in the private patients than in the paupers; indeed, the latter show a lower rate of increase than usual.

Of the total number we find that 2,532 were maintained from private sources, 14,309 by parochial rates, and 53 at the expense of the State.

It is far from pleasing to be told every year and in every division of the United Kingdom that there is a steady rise in the number of the registered insane; and although Scotland only records an increase of 236, it is more significant when read with the knowledge that the total increase of the insane since 1858 has been 11,070, and that the number of lunatics has increased 190 per cent., while the increase of the population during the same period has only been 52 per cent. Further it is pointed out that the number of private patients has increased by 111, and that these cases have now reached the proportion of 49 to every 100,000 of the population. The Report states that a considerable number of these private patients are sent from the English side of the border; a fact which shows that great confidence exists in the Scottish lunacy system, a confidence which we ourselves share; but nevertheless we should like to know what steps are being taken to check this terrible influx of lunatics to our asylums, and to reduce the burden from the shoulders of the ratepayer ere such burden becomes intolerable.

The percentage of recoveries has a wide range. It is 6 per cent. in the lunatic wards of poorhouses and 50 per cent. in parochial asylums, while royal and district asylums show 39 per cent. of cures, and private asylums 40 per cent. The Commissioners are careful to remark that very erroneous inferences might be drawn from these percentages unless the nature of the cases admitted to the various institutions be kept in view. The death-rate was 9·5 per cent. of the average number resident.

During the year 1903 there were 168 patients escaped from the asylums of Scotland. If captured within 28 days fresh certificates are not required for their confinement, but of the escapes in 1903 there were 20 who managed to make good their escape and were struck off the books. Five of these were said to have recovered, but we doubt whether a perfectly sane patient ever attempted to escape when awaiting his discharge. We are glad to see that the proportion of escapes is a diminishing one and that in ten years it has fallen from 18 per 1,000 to 12 per 1,000.

Regarding the condition of establishments for the insane in Scotland, it is evident that much good work has been done during the year 1903. Among others we notice that a new villa for male patients has been opened at the Banff Asylum; a sanatorium for consumption has been erected at Dumfries; the Glasgow Asylum is rendering a beneficial service by receiving patients in straitened circumstances at considerably reduced rates of board; a sanatorium for 60 patients has been opened at the Gartloch Asylum. At the Woodilee Asylum the proportion of day attendants to patients is 1 to 8, and of night attendants 1 to 32. The Commissioners say that this proportion is not exceeded by any institution in the country and we readily accept the statement; but we should like to ask the Commissioners whether they consider such a proportion necessary, and if not whether they think it justifiable in a rate-supported asylum. We learn that in the Murray Asylum at Perth there are 40 patients who pay an average of £45 a year for board, which is about half the ordinary minimum for private patients, and it stamps the institution as a veritable charity. Long may it continue so and shame those sister asylums which are far too much on the outlook for high paying cases.

Other information to be culled from this blue book is not always so satisfactory. For instance, the Roxburgh Asylum contains 44 patients in excess of its proper number, and the Haddington Asylum has 22 more than its space is intended for.

There were nine deaths from suicide during the year, being about 1 to 1,800. This proportion is more than twice as great as it was in English asylums during 1902.

The Commissioners are strongly of opinion that district lunacy boards should be allowed to provide accommodation for private patients of the poorer class; and it is difficult to understand how there could be any other opinion on the question, it being understood that the provision should be plain and the rates charged be just above the maintenance rate for paupers.

